# Domestic Summary

**Published:** 1838-01-03

**Authors:** 


### DOMESTIC SUMMARY.

### LITHOTRIPSY.

The November number of the American Journal of the Medical Sciences contains an account of four successful lithotriptic operations by Dr. J. Randolph, of this city. Dr. R.'s triumph in this department of surgery, on these and former occasions, has been signal and complete.

In the same Journal Dr. N. R. Smith of Baltimore, the late Professor of Surgery in the University of Maryland, reports six cases of lithotripsy, of which four were performed on children; two of whom were about seven, and the remainder infants. The most flattering success attended all his efforts; he has most satisfactorily proved that it is an operation as equally well adapted to children as to adults. Dr. Smith expresses his decided preference of the *brise-pieere* of Jacobson over Baron Hourteloup's *lithontripteur;* in this respect his experience corresponds with that of Dr. Randolph. Contrary to the received practice, Dr. S. operated with the bladder empty and without the slightest injury to that viseus, whilst he thinks the grasping of the stone facilitated.

In the clinical surgery reports of the Philadelphia Hospital, (Blockley,) by Professor Horner, at p. 108 of the same Journal, a fortunate case of lithotripsy is recorded. "The procedure of this operation was partly by striking with the hammer, and partly by screwing up the instrument," (which was Hourteloup's,) "the bladder having been previously filled with four ounces of tepid barley water."

---

### AUTOPLASTY.

The Taliacotian operation, revived and modified by Dieffenback of Berlin, has been lately attempted in this country. Dr. J. Mason Warren, of Boston, succeeded admirably with a case occurring in his practice, last winter. Dr. Mütter, of this city, lately performed a rhinoplastic operation with the happiest results. One of the alœ nasi being destroyed, Dr. M. availed himself of the known extensibility of the integument of the cheek, and replaced the lost portion, by dissecting up a flap from the adjacent soft parts and applying it to the freshened edges of the ulcer. By this method the deformity of a cicatrix and the uncertainty of re-establishing the circulation were avoided. The patient left the city a few weeks since with his appearance entirely changed and renovated—no vestiges of the operation were apparent. This is Dr. M.'s second successful effort at autoplasty. In the August number of the American Journal of the Medical Sciences, he reports an interesting and fortunate instance of genioplasty. Dr. Mütter has devoted considerable attention to this department of surgery.

### HYSTEROTOMY.

Dr. Gibson, Professor of Surgery in the University of Pennsylvania, has been successful a second time with this operation, upon the same woman. It was performed on Sunday, November 5th, ten hours after labor had commenced. The child's head presented at the inferior strait, and the membranes were ruptured. Some difficulties were experienced in extracting the foetus, owing to the sudden and violent contractions of the uterus; this was, however, successfully accomplished by Dr. C. D. Meigs. We saw the patient a few days after the operation, through the politeness of Dr. Geo. Fox, under whose care she was, and to whose judicious management of the after-treatment, much of the ultimate success may be attributed. She was doing very well, and the wound healing rapidly. She told us she had suffered much less than when delivered *per vias naturales.* It is now eight weeks since its performance, and the woman has perfectly recovered, without a single bad symptom. The infant is in excellent condition.

This is an additional proof of the propriety of prompt action, when this operation is once determined upon. For a very interesting account of this woman's first labor, when cephalotomy was performed, see North Am. Med. and Surg. Journal, No. xxiv. Oct. 1831, communicated by Dr. Fox.

